# Transcriptomic signatures in trophectoderm and inner cell mass of human blastocysts classified according to developmental potential, maternal age and morphology

**DOI:** 10.1371/journal.pone.0278663

**Published:** 2022-12-01

**Authors:** Yoshiteru Kai, Hailiang Mei, Hiroomi Kawano, Naotsuna Nakajima, Aya Takai, Mami Kumon, Azusa Inoue, Naoki Yamashita

**Affiliations:** 1 Reproductive Medicine Research Center, Yamashita Shonan Yume Clinic, Fujisawa, Japan; 2 RIKEN Center for Integrative Medical Sciences, Yokohama, Japan; 3 Tokyo Metropolitan University, Hachioji, Japan; Utah State University, UNITED STATES

## Abstract

Selection of high-quality embryos is important to achieve successful pregnancy in assisted reproductive technology (ART). Recently, it has been debated whether RNA-sequencing (RNA-Seq) should be applied to ART to predict embryo quality. However, information on genes that can serve as markers for pregnant expectancy is limited. Furthermore, there is no information on which transcriptome of trophectoderm (TE) or inner cell mass (ICM) is more highly correlated with pregnant expectancy. Here, we performed RNA-Seq analysis of TE and ICM of human blastocysts, the pregnancy expectation of which was retrospectively determined using the clinical outcomes of 1,890 cases of frozen-thawed blastocyst transfer. We identified genes that were correlated with the expected pregnancy rate in ICM and TE, respectively, with a larger number of genes identified in TE than in ICM. Downregulated genes in the TE of blastocysts that were estimated to have lower expectation of pregnancy included tight junction-related genes such as *CXADR* and *ATP1B1*, which have been implicated in peri-implantation development. Moreover, we identified dozens of differentially expressed genes by regrouping the blastocysts based on the maternal age and the Gardner score. Additionally, we showed that aneuploidy estimation using RNA-Seq datasets does not correlate with pregnancy expectation. Thus, our study provides an expanded list of candidate genes for the prediction of pregnancy in human blastocyst embryos.

## Introduction

In assisted reproductive technology (ART), cleavage-stage embryos (2–3 days post-fertilization) and blastocysts (5–6 days post-fertilization) are generally used for embryo transfer, with the latter associated with a higher rate of pregnancy [1]. With the advent of time-lapse imaging systems, temporal morphological assessment is currently the primary method for embryo quality evaluation [2]. Reportedly, the degree of blastocoele expansion and re-expansion correlates with the rate of live births in fresh and frozen-thawed blastocyst transfer cycles [3, 4]. In addition, time-lapse monitoring revealed that blastocyst collapse *in vitro* correlates with implantation and pregnancy outcomes [5]. Thus, temporal morphological parameters are considered beneficial for increasing pregnancy outcomes.

Nonetheless, it remains controversial whether the morphology of trophectoderm (TE) or inner cell mass (ICM) correlates better with pregnancy outcomes. Several studies have reported that the morphology of TE is more correlated with pregnancy outcomes than that of ICM [6–9]. For example, Hill et al. [7] conducted a retrospective analysis of pregnancy outcomes of 694 frozen-thawed blastocysts. They showed that the grading of TE, but not that of ICM, was significantly correlated with the rates of implantation and live birth. In contrast, other studies have revealed that ICM correlates better with pregnancy outcomes than TE [10, 11]. For example, Richter et al. [10] conducted a prospective observational study with 178 blastocysts and revealed that quantitative measurements of the size and shape of ICM were highly indicative of implantation potential, whereas the cell numbers of TE were unrelated to implantation rates. Therefore, it is essential to gain molecular insights into the ICM and TE of blastocysts using pregnancy expectation estimation.

Pre-implantation genetic testing for aneuploidy (PGT-A) has also been used in clinical practice to prevent miscarriages in patients with unexplained recurrent pregnancy loss, as it can detect aneuploid embryos. However, the inefficiency of PGT-A has been demonstrated in recent studies considering the mosaicism of embryos and the heterogeneity of biopsy techniques among clinics [12–14]. Additionally, embryo morphokinetics cannot accurately reflect the degree of chromosomal mosaicism [15]. Even diploid embryos with normal morphological parameters may result in miscarriages [16].

The problems underlying the morphological parameters and PGT-A for blastocyst selection may be complemented by transcriptome analyses. A few studies have reveal a correlation of gene expression data of TE of human blastocysts with pregnancy [17–20]. Jones et al. [17] conducted a microarray analysis of biopsied TE of pooled viable and non-viable blastocysts. However, these data do not establish a direct correlation between gene expression and the implantation rate of individual embryos. Kirkegaard et al. [18] and Ntostis et al. [19] conducted RNA-sequencing (RNA-Seq) of biopsied TE of individual blastocysts. The embryo quality was prospectively estimated based on the outcome of live births. They identified several genes significantly differentially expressed between embryos with pregnancy failure and those with success. Groff et al. [20] performed RNA-Seq analysis to compare biopsied TEs and the remaining whole embryos to demonstrate that biopsied TEs can capture valuable information, including RNA-based digital karyotypes and several candidate genes associated with morphological superiority. Although these studies are helpful, information on genes that can serve as markers for pregnancy expectancy is limited. In this study, we examined the correlation between expected pregnancy rate, gene expression profiles of ICM and TE, and karyotypes.

## Materials and methods

### Institutional review board approval

This study was approved by the Ethical Review Committees of the National Center for Global Health and Medicine and RIKEN Center for Integrative Medical Sciences for both collection and experimental use of surplus blastocysts obtained from infertility treatment and donated for research (approval numbers: NCGM-A-003371-00 and 2020–8). It was registered at the Japan Society of Obstetrics and Gynecology (approval number: 195).

### Consent process

This study was performed in accordance with the tenets of the Declaration of Helsinki. All blastocysts used in this study were donated and stored at Yamashita Shonan Yume Clinic for research purposes. Couples who had blastocysts derived from c-IVF or ICSI donated surplus blastocysts for research after written/verbal informed consent and signed an extensive consent form for inclusion in the study. These consent forms were approved by the Ethical Review Committees of the National Center for Global Health. We did not have access to any personal health information of the couples.

### Blastocyst evaluation correlated with the pregnancy rate

**Fig 1:**
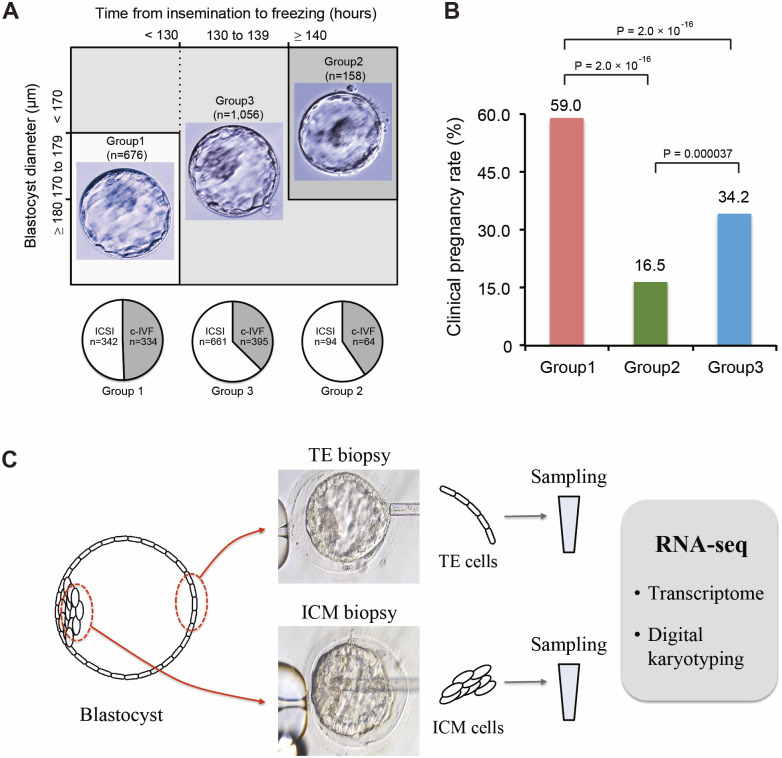
Overview of blastocyst classification and biopsy. (A) A classification based on the diameter and growth rate of the blastocysts utilized for embryo transfer. Representative images of a blastocyst from each group, and the number of implanted blastocysts are shown. Three pie charts represent the insemination method for each group. (B) Clinical pregnancy rates of each group. (C) Biopsy procedure for RNA-Seq. Trophectoderm (TE) samples and an inner cell mass (ICM) samples were collected from each blastocyst and processed for RNA-Seq.

Blastocysts were classified according to their development rate and diameter, as described in Fig 1A. In addition, Gardner scores are shown in Table 1 for comparison with conventional blastocyst evaluation methods [21]. The correlation between blastocyst evaluation and pregnancy rate was retrospectively analyzed in 1890 cases subjected to vitrified/warmed blastocyst transfer between March 2018 and December 2020 at Yamashita Shonan Yume Clinic. Pregnancy was defined as confirmed presence of gestational sac (GS).

**Table 1:** List of blastocysts used for RNA-seq.

	Sample ID	Maternal age at oocytes retrieval	Fertilization procedure	Gardner score	Number of sampling cells	Number of oocytes collected in the cycle	Number of frozen blastocysts in the cycle
**Group1**	H1	31	ICSI *	4BB	ICM: 10~15 cells, TE: 15~20 cells	11	5
	H2	42	c-IVF	4AA	ICM: 10~15 cells, TE: 10~15 cells	3	2
	H3	37	c-IVF	4AB	ICM: 5~10 cells, TE: 10~15 cells	6	5
	H4	29	c-IVF	4BB	ICM: 5~10 cells, TE: 10~15 cells	4	2
	H5	28	ICSI	4BB	ICM: 5~10 cells, TE: 10~15 cells	2	2
**Group2**	L1	38	ICSI	4CC	ICM: 10~15 cells, TE: 10~15 cells	6	2
	L2	37	ICSI	4CC	ICM: 15~20 cells, TE: 10~15 cells	2	1
	L3	39	ICSI	4CC	ICM: 30~40 cells, TE: 10~15 cells	5	4
	L4	39	ICSI	4CC	ICM: 30~40 cells, TE: 10~15 cells	4	2
**Group3**	M1	40	c-IVF	4CB	ICM: 15~20 cells, TE: 10~15 cells	5	2
	M2	33	c-IVF	4CC	ICM: 10~15 cells, TE: 10~15 cells	2	1
	M3	39	ICSI	4CC	ICM: 30~40 cells, TE: 10~15 cells	1	1
	M4	37	c-IVF	4BB	ICM: 30~40 cells, TE: 15~20 cells	1	1

* ICSI after oocyte activation treatment with electrical stimulation,

** All blastocysts were donated by different patients for this study

### Blastocyst classifications by Gardner score and maternal age

Blastocysts were classified by the Gardner score [21]. Those with Gardner scores of BB or higher were classified as “good” group (H1-5 & M4), and the others were classified as “poor” group (Table 1). In addition, blastocysts obtained from patients younger than 35 years were classified as “young” group (H1, H4, H5 & M2), and those obtained from patients elder than 35 years of age were classified as “elder” group (H2-3, M1, M3-4 & L1-4) (Table 1).

### Biopsy

Biopsy was performed on the heated stage of an IX-73 microscope (Olympus, Japan), equipped with a micromanipulator (Narishige Group, Japan) and an infrared diode laser (Saturn 5TM Laser system; CooperSurgical, Inc., Japan), in dishes prepared with droplets of HEPES-buffered calcium/magnesium-free medium (Sage In-Vitro Fertilization, Inc., USA) and biopsy medium (1062; Origio, Denmark). A diode laser was used to assist the opening of a 10–20-μm hole in the zona pellucida using 2–4 laser shots. Biopsies were performed using a glass pipette (PIN20-20FT; PrimeTech Corporation, Japan). ICM biopsies were performed either by laser removal of a small area of the TE layer opposite the ICM to aspirate ICM cells with a biopsy pipette or directly introducing the biopsy pipette into the blastocoel cavity to aspirate ICM cells. The TE biopsy was performed either by aspirating the TE layer that was removed by the laser during the ICM biopsy or collecting a new sample from the TE layer after removal of the ICM. In the biopsies used in this study, 5–40 cells were collected from both ICM and TE. The biopsy samples were transferred to a polymerase chain reaction (PCR) tube (FG-021F; NIPPON Genetics Co., Ltd., Japan) containing 0.5 μL of phosphate-buffered saline (PBS) and frozen. The frozen samples were stored at -80°C until RNA-Seq.

### Library preparation and sequencing

RNA-Seq libraries of all biopsy samples were generated using a SMART-Seq HT Kit according to the manufacturer’s instructions (TAKARA, 634436, Japan). Library construction was performed using a Nextera XT DNA Library Prep Kit (Illumina, USA) with 200 pg of cDNA. The libraries were sequenced on NextSeq 500 with single-end 75-bp reads (Illumina).

### Transcriptome analysis

All sequencing reads were mapped to the human reference genome (hg19) using STAR (version 2.7.6a) with default parameters after evaluating the quality using FastQC (version 0.11.9) and trimming low-quality bases or adapter sequences using TrimGalore (version 0.6.4). RSEM (version 1.3.1) was used to calculate the gene expression level using the transcripts per million (TPM) value. To construct a high-quality reference transcriptome for downstream analyses, genes with TPM values greater than 1 in one or more samples were regarded as expressed genes. Genes with TPM values less than 1 in all samples were filtered out. For transcriptome coverage analysis, genes with TPM values greater than 1 in 6 or more samples were used as the final filtered reference set. The number of expressed genes in the ICM and TE were 10 605 and 11 119, respectively. The coverage of each sample was defined as the proportion of genes expressed in the final reference set.

To identify differentially expressed genes (DEGs), featureCounts (version 2.0.0) was used to calculate the read count per gene. Thereafter, DEGs (q-value < 0.01) were identified using the R package “DESeq2” (version 1.32.0) which used a negative binomial distribution to model the RNA-seq counts and developed a shrinkage estimation for dispersions and fold changes to improve stability. The Gene Ontology (GO) term enrichment analysis was conducted using Metascape.

### Digital karyotyping

According to Groff et al. [20], the Z-score approach was applied to assess RNA-based digital karyotypes of sex chromosomes and autosomes. To determine the copy number of the X chromosome, all reads mapped to the expressed genes on the X chromosome (except for genes located in pseudoautosomal regions 1 and 2) were summed per sample and normalized for sequencing depth. The Z-score was calculated across the ICM and TE samples. The mean and SD of the normalized read count were calculated across all samples per sample type (TE or ICM). Positive and negative Z-scores indicated the presence of two copies and one copy of the X chromosome, respectively. Furthermore, to examine the existence of the Y chromosome, the TPM values of three genes (*ddx3y*, *rps4y1*, and *eif1ay*) located on the Y chromosome were summed, and a total of >250 TPMs provided a clear indication of the existence of the Y chromosome. To predict the copy number of each autosome, the Z-score cutoff was set at ±2, and the out-layered chromosomes were identified as potential aneuploids, as described previously [20].

### Statistical analysis

The chi-square with Bonferroni multiple comparison test was performed to identify potential relationships between morphological parameters of the vitrified blastocyst and clinical pregnancies. Statistical significance was set at p < 0.001. These statistical tests were conducted using EZR [22].

## Results

### Classification of blastocysts based on the retrospective analysis of the pregnancy rate

The blastocyst development rate and blastocyst diameter at a given time point can be good parameters for predicting pregnancy outcomes [3, 23–25]; therefore, we retrospectively analyzed the clinical outcomes of 1890 cases of vitrified-warmed blastocyst transfer performed in our clinic. Based on the diameter and the time period to reach the blastocyst stage, the embryos were classified into three groups (Fig 1A): those that had taken less than 130 h to reach a diameter of >170 μm (Group 1, n = 676), those that had taken more than 140 h to reach a diameter of < 180 μm (Group 2, n = 158), and those of the rest (Group 3, n = 1,056)” (Fig 1A). The pregnancy rates in Groups 1, 2, and 3 were 59.0%, 16.5%, and 34.2%, respectively (p < 0.001) (Fig 1B). Given this high correlation with pregnancy outcome, we hereinafter regarded Groups 1, 2, and 3 blastocysts as high, low, and intermediate expectations of pregnancy, respectively.

### Validation of RNA-Seq datasets

We used 5 blastocysts with high expectations of pregnancy (H1–H5), 4 with intermediate expectations of pregnancy (M1–M4), and 4 with low expectations of pregnancy (L1–L4) for RNA-Seq analysis (Fig 1C, Table 1). These blastocysts were derived from separate patients (Table 1). To understand which transcriptomes of ICM or TE shows a greater correlation between their transcriptomes and the expectation of pregnancy, we dissected blastocysts into ICM and TE and performed RNA-Seq (S1 Table).

First, we determined the quality of the RNA-Seq data. Principal component analysis (PCA) confirmed that ICM and TE samples were well segregated in all groups, validating the successful dissection of ICM and TE (S1A Fig). We further confirmed that the number of expressed genes (TPM > 1) was approximately 10 000 in both ICM and TE samples in all groups (S1B Fig). In addition, plotting the number of expressed genes and transcriptome coverage confirmed that the complexity of the RNA-Seq library of L1–L4 was not lower than that of H1–H5 (S1C and S1D Fig). These results exclude the possibility that blastocysts with lower expectations of pregnancy have an aberrant quantity of whole transcripts in the ICM or TE.

### Digital karyotyping using RNA-Seq datasets

**Fig 2:**
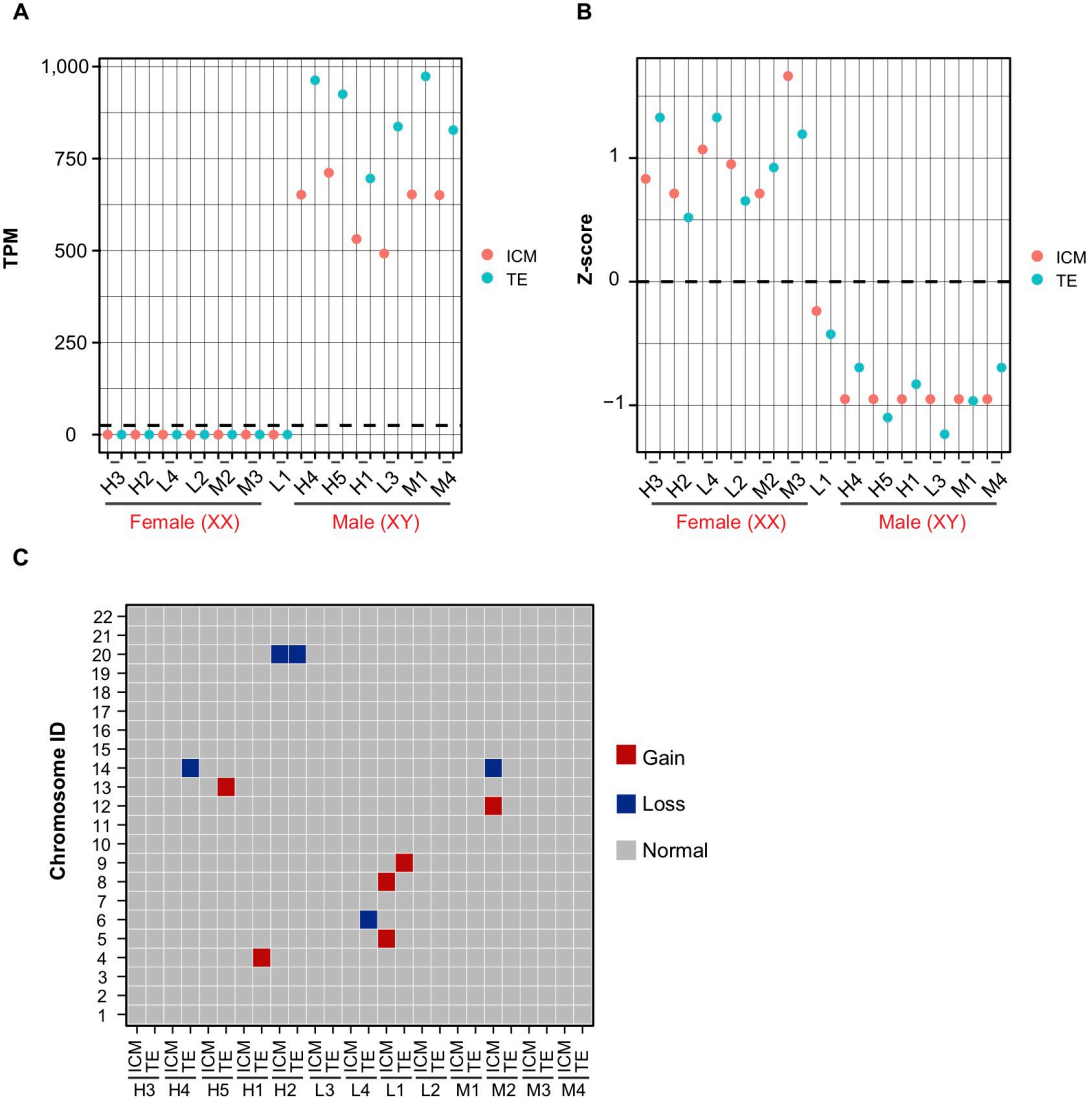
Digital karyotyping using RNA-Seq. (A) Sum of TPM values for genes on the Y chromosome. Sexes in red letters are the predictions. (B) Z-score profiles of gene expression levels of X chromosome-linked genes. Sexes in red letters are the predictions. (C) Digital karyotyping results. Gray indicates estimated euploidy with Z-score ± 2. Red indicates the estimated gain of the chromosome (Z-score > 2). Blue indicates an estimated loss of chromosome (Z-score < -2). This cutoff was according to a previous study [20].

To examine whether lower expectations of pregnancy involve abnormalities in karyotypes, we conducted digital karyotyping using RNA-Seq datasets. We first checked the expression levels of Y chromosome-linked genes to determine the embryo sex. Y-linked genes were expressed in 12 of the 26 samples (Fig 2A). The 12 samples comprised six pairs of ICM and TE samples from single blastocysts, indicating XY embryos. Next, we analyzed X chromosome-linked genes using a Z-score approach to estimate the copy numbers of X chromosomes, as described in a previous study [20]. The expression of X-linked genes was higher in 12 of the 26 samples (Fig 2B). These 12 samples again comprised six pairs of ICM and TE samples from single blastocysts, indicating that they were XX embryos. We noticed that X-linked genes were intermediately expressed in L1 embryos (Fig 2B). As the L1 embryo did not express Y-linked genes, we speculated that this embryo might be XO, which lacks one of the X chromosomes. Next, to estimate the karyotypes of the autosomal chromosomes, we performed RNA-based digital karyotyping. Based on a previous study [20], we considered chromosomes with Z-scores less than ±2 as euploid, those with Z-score more than 2 as a potential gain of chromosomes, and those with Z-score less than -2 as potential loss of chromosomes. Among the five blastocysts with high expectations of pregnancy (H1 to H5), four showed potential gain or loss of at least one chromosome in either ICM or TE (Fig 2C). In four blastocysts with intermediate (M1 to M4) and low expectations of pregnancy (L1 to L4), only 1 (M2) and 2 (L1 and L4) showed potential gain or loss of at least one chromosome in either ICM or TE, respectively (Fig 2C). Thus, these findings suggest that RNA-based digital karyotyping alone does not allow the prediction of pregnancy expectations.

### Identification of genes associated with pregnancy expectation

**Fig 3:**
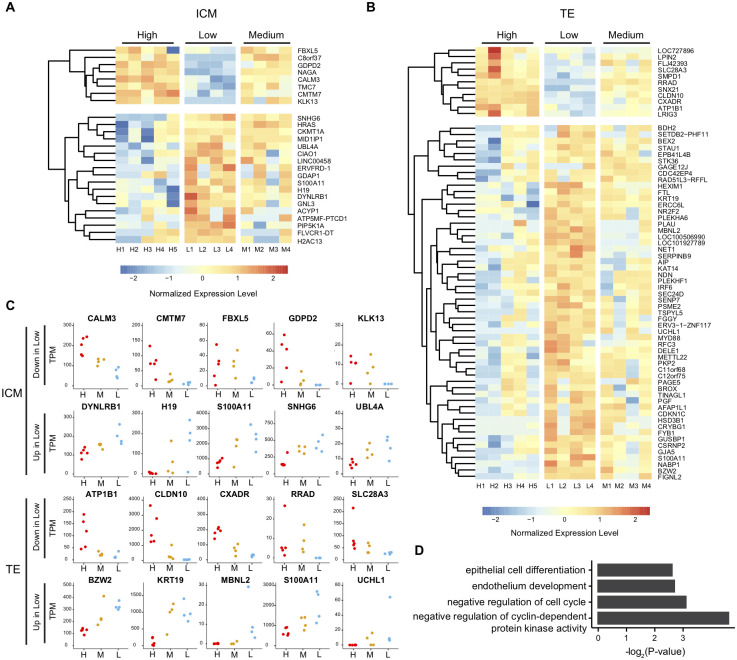
Identification of differentially expressed genes (DEGs). (A, B) Heatmap showing the relative expression level of DEGs in ICM (A) and TE (B). (C) Jitter plots of expression level (TPM) in representative DEGs. (D) Gene Ontology term enrichment of upregulated DEGs in TE of blastocysts with a low expectation of pregnancy.

Having validated the quality of our RNA-Seq datasets, we attempted to identify genes associated with the expectation of pregnancy. Using the criteria of q-value < 0.01 (calculated by DESeq2) comparing H1 to H5 and L1 to L4, we identified 141 and 263 DEGs in ICM and TE, respectively. To obtain the most reliable genes, we filtered out genes with high variability between individual blastocysts in each group by calculating the Z-score among all samples. The reliable DEGs were picked if the deviation of Z-score between H1 to H5 and L1 to L4 was less than 0.5. Consequently, 26 and 67 DEGs were identified as the most reliable DEGs in the ICM and TE, respectively (S2 and S3 Tables). Among the 26 DEGs identified in ICM, eight showed higher expression in blastocysts with high expectations of pregnancy than those with low expectations, and 18 showed the opposite trend (Fig 3A). Among the 67 DEGs identified in TE, 11 showed higher expression in blastocysts with high expectations of pregnancy than those with low expectations, and 56 showed the opposite trend (Fig 3B). Jitter plots for the gene expression levels (TPM) of the five representative DEGs in each group are shown (Fig 3C). The blastocysts with the intermediate expectation of pregnancy (M1 to M4) showed intermediate expression levels of these DEGs (Fig 3A–3C), which further supported the idea that these DEGs could serve as valuable markers for pregnancy expectation. We performed GO term enrichment analysis of the 56 DEGs whose expression levels were higher in the TE of blastocysts with lower expectations of pregnancy. The results demonstrated that biological functions related to epithelial cell differentiation and negative regulation of the cell cycle were enriched (Fig 3D), suggesting that excessive differentiation of epithelial cells and cell cycle defects in TE might cause pregnancy failure. The number of DEGs in the ICM was too small to perform GO analysis.

### Identification of differentially expressed genes according to the maternal age and Gardener score

**Fig 4:**
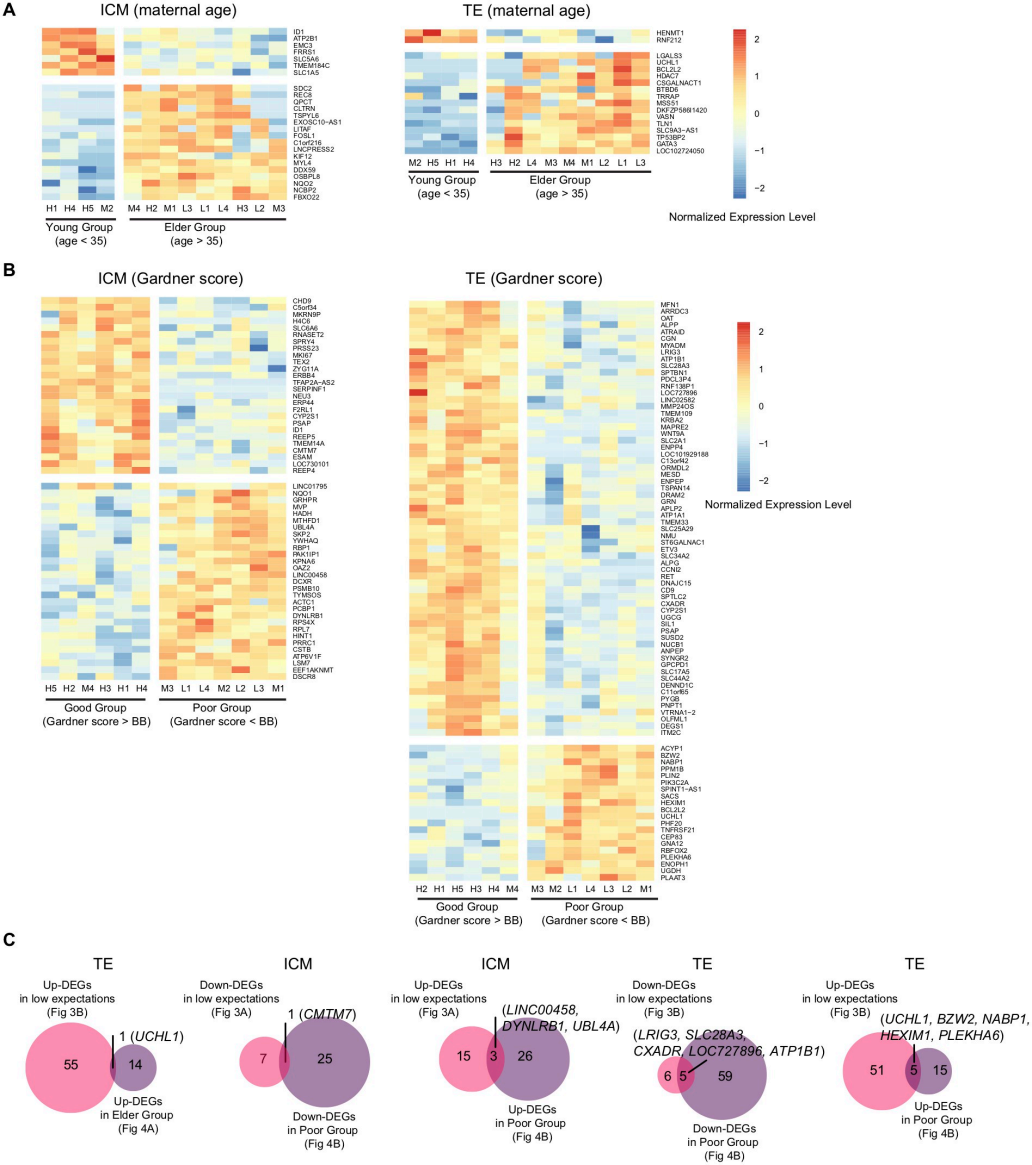
Comparison of differentially expressed genes (DEGs) based on the maternal age and Gardner score. (A, B) Heatmap showing the relative expression level of DEGs identified by classification of the maternal age (A) and the Gardner score (B). (C) Venn diagram displaying common DEGs when comparing the expectations of pregnancy with the maternal age and the Gardner score. Although there are theoretically eight patterns of comparison, only 5 patterns of comparison were indicated because no common genes were found in the other three patterns.

We then examined whether common DEGs could be found when the RNA-seq data were classified by the maternal age and Gardner score, respectively. When classified by the maternal age (>35 versus < 35) (Table 1), we identified 7 and 17 genes that were down- and up-regulated in the elder group, respectively, in ICM. In TE, 2 and 12 genes were down- and up-regulated in the elder group, respectively (Fig 4A and S4 Table). When classified by Gardner score (<BB versus <BB) (Table 1), we identified 26 and 29 genes that were down- and up-regulated in the poor group, respectively, in ICM. In TE, 64 and 20 genes were down- and up-regulated in the poor group, respectively (Fig 4B and S5 Table). Comparison of these genes and DEGs identified by the pregnancy expectation found 14 common genes (Fig 4C). By comparison with the elder group, we identified one gene (*UCHL1*) that was commonly up-regulated in TE. By comparison with the poor Gardner score group, one gene (*CMTM7*) was commonly down-regulated and three genes (*LINC00458*, *DYNLRB1 and UBL4A*) were commonly up-regulated in ICM, and 5 genes (*LRIG3*, *SLC28A3*, *CXADR*, *LOC727896 and ATP1B1*) were commonly down-regulated and 5 genes (*UCHL1*, *BZW2*, *NABP1*, *HEXIM1 and PLEKHA6*) were commonly up-regulated in TE (Fig 4C).

## Discussion

RNA-Seq of human blastocysts has advanced our fundamental understanding of the molecular mechanisms underlying human preimplantation development [26–29]. Previous studies have linked the transcriptomes of biopsied TE obtained using RNA-Seq with the birth ratio after embryo transfer [18–20]. Nevertheless, information on genes that can serve as markers of pregnancy expectancy is still limited. In addition, it remains unknown which transcriptomes of TE or ICM are more strongly correlated with pregnancy expectancy. In the present study, we used the clinical pregnancies of 1890 cases to establish novel criteria for evaluating pregnancy potential of blastocysts and identified several candidate genes that might be useful for pregnancy estimation. We also found that the number of DEGs in TE was higher than that in ICM, suggesting that genes expressed in TE, rather than ICM, might contribute more to the peri-implantation period and that TE provides a better chance to predict pregnancy outcomes.

Although PGT-A is considered to increase the implantation rate, recent reports have argued against the positive effects of PGT-A [30–32]. Digital karyotyping using our RNA-Seq datasets allowed us to determine whether aneuploidy in blastocysts was correlated with pregnancy expectancy. The results did not show a correlation between the estimated karyotypic abnormalities and lowered expectation of pregnancy, supporting the notion that investigation of aneuploidy alone with PGT-A may not be sufficient to select high-quality embryos for transfer. Therefore, more comprehensive evaluations using simple karyotyping should contribute to the prediction of embryo quality.

The present study provides a list of genes that are potentially correlated with the expectation of pregnancy. We further identified DEGs by the maternal age and morphological grading (Gardner score). These results suggested that the classification of blastocysts based on the retrospective analysis of pregnancy rates is better correlated with Gardner score than with maternal age. CKLF-like MARVEL transmembrane domain-containing 7 (*CMTM7*), which was identified as a common DEG in ICM, is known to be important for activation of downstream signaling pathways of ERK [33]. Since ERK signaling has been reported to be important in the development of three cell lineages in mice [34], *CMTM7* might play a role in preimplantation embryo development. Ubiquitin C-terminal hydrolase L1 (*UCHL1*) in TE was found to be commonly up-regulated in both of the elder maternal age and the poor Gardner score. Notably, *Uchl1* is known to be essential for mouse blastocyst development [35]. Although the function of human *UCHL1* remains unknown, it might serve a marker for blastocyst quality. CXADR Ig-like cell adhesion molecule (*CXADR*) and ATPase Na^+^/K^+^ transporting subunit beta 1 (*ATP1B1*) were identified as commonly down-DEGs in the TE of blastocysts with low expectations of pregnancy and with the poor Gardner score. These genes are known to be essential for human or mouse development. *CXADR* regulates tight junction stability, and its knockdown reduces implantation potential owing to tight junction defects in mice [36]. *ATP1B1* plays a vital role in forming tight junctions in the trophoblast membrane of mice [37]. The uptake of Na^+^ ions into the blastocoel cavity by Na/K-ATPase participates in the expansion of human blastocysts [38]. A lack of tight junctions or loss of function of the Na/K-ATPase pumps leads to a decrease in lumen size and cortical tension of trophectoderm cells [39]. Thus, *CXADR* and *ATP1B1* likely play essential roles in development via the regulation of blastocyst collapse.

This study had a limitation. Although we established an expected pregnancy rate in relation to the degree of blastocyst development from retrospective clinical outcomes and used it as a surrogate marker for assigning biopsied blastocysts to different analysis groups, full-term development of the blastocysts used for RNA-Seq were not monitored and the sample size was limited. Therefore, it remains unknown whether the gene expression profiles accurately reflects the pregnancy outcomes. Nevertheless, our findings provide candidate genes potentially helpful in establishing non-invasive selection of high-quality blastocysts for ART. Future studies should reveal the detailed functions of these genes and determine whether genes identified in this study participate in human development. As in vitro implantation models of human embryos and artificial blastoids have recently been developed, these would contribute to understanding the functions of the genes in the near future [40, 41].

## Conclusions

This study revealed that classification based on the rate of blastocyst development and blastocyst diameter is highly correlated with pregnancy rate, and further identified several genes differentially expressed by RNA-Seq using the classified blastocysts. Furthermore, we identified 14 DEGs commonly identified by regrouping blastocysts according to the maternal age and Gardner score. A digital karyotype was created from the number of RNA reads concurrently with gene expression analysis, but we did not observe a correlation between the predicted karyotype aberrations and the predicted developmental potential. These results are not only expected to be utilized in the future, such as in the establishment of a scientifically based method for non-invasive selection of blastocysts with the potential for successful pregnancy in ART, but will also be useful for basic research on transcriptional events in human pre-implantation development.

## Supporting information

S1 Fig Quality assessment for RNA-Seq samples. (A) Principal component analysis (PCA) of gene expression level in all embryo samples. ICM and TE are shown in two different shapes. The grades of embryo quality are shown in three colors. (B) Number of genes with expression levels greater than one was measured using transcripts per million (TPM) in ICM and TE. (C) Box plot showing the number of genes with TPM greater than 1. (D) Percent coverage of the total expressed genes in ICM and TE. The expressed genes were defined by TPM greater than 1 in at least one embryo sample.
(TIF)

S1 Table FPKM of all genes. (XLSX)

S2 Table List of DEGs in ICM. (XLSX)

S3 Table List of DEGs in TE. (XLSX)

S4 Table List of DEGs according to the maternal age. (XLSX)

S5 Table List of DEGs according to the Gardner score. (XLSX)
