# Supplementary figures and images for: Transcriptomic signatures in trophectoderm and inner cell mass of human blastocysts classified according to developmental potential, maternal age and morphology
Source: PLoS One. 2022 Dec 1;17(12):e0278663. doi: 10.1371/journal.pone.0278663 (PMC9715016; doi:10.1371/journal.pone.0278663)

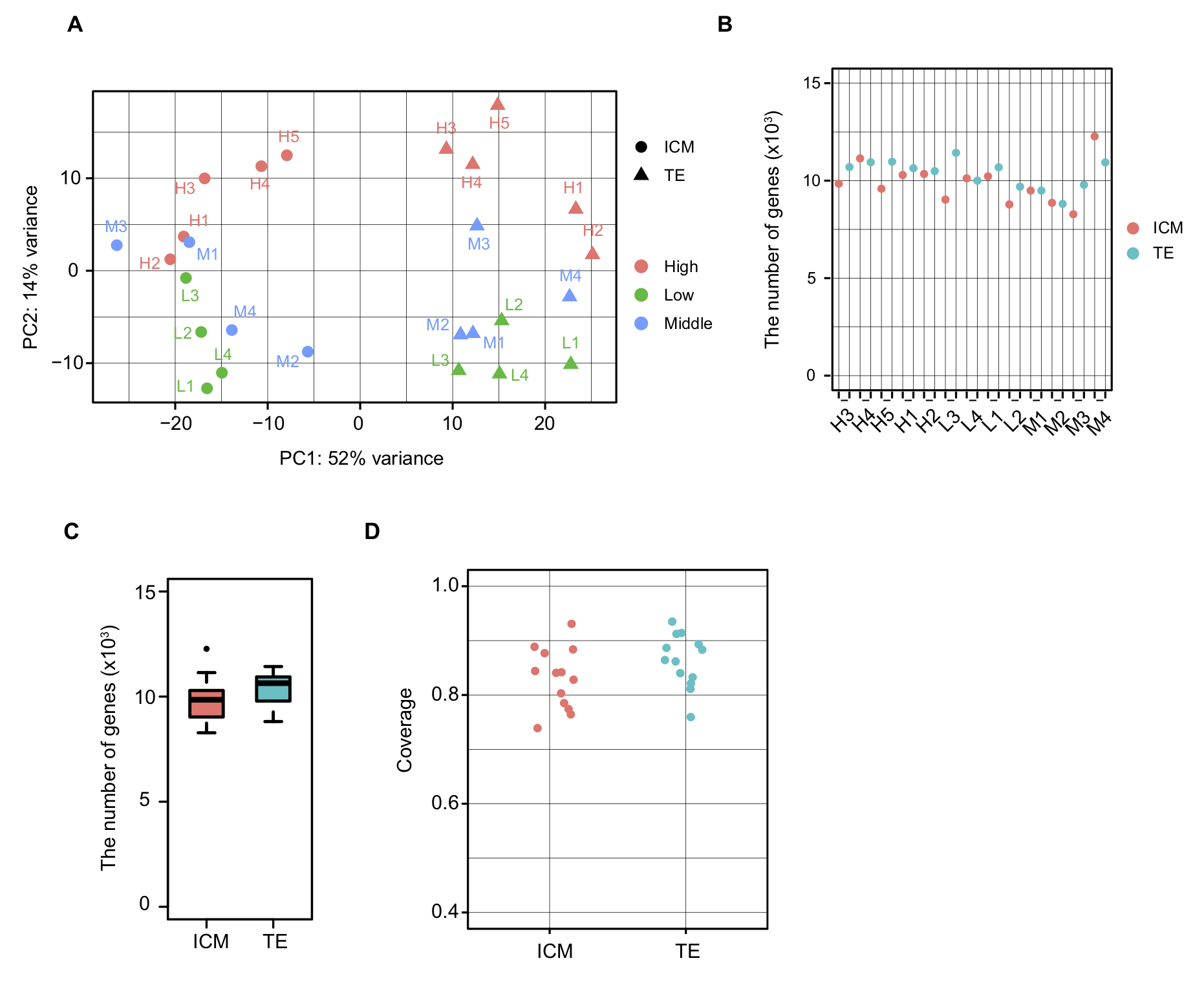

Supplement: S1 Fig — (A) Principal component analysis (PCA) of gene expression level in all embryo samples. ICM and TE are shown in two different shapes. The grades of embryo quality are shown in three colors. (B) Number of genes with expression levels greater than one was measured using transcripts per million (TPM) in ICM and TE. (C) Box plot showing the number of genes with TPM greater than 1. (D) Percent coverage of the total expressed genes in ICM and TE. The expressed genes were defined by TPM greater than 1 in at least one embryo sample. (TIF) [file pone.0278663.s001.tif]
